# The devil is in the detail: reflections on the value and application of cognitive interviewing to strengthen quantitative surveys in global health

**DOI:** 10.1093/heapol/czab048

**Published:** 2021-06-25

**Authors:** K Scott, O Ummer, A E LeFevre

**Affiliations:** Department of International Health, Johns Hopkins Bloomberg School of Public Health, 615 N. Wolfe Street, Baltimore, MD 21205, USA; Oxford Policy Management, 4/6 1st Floor, Siri Fort Institutional Area, 11049 New Delhi, India; BBC Media Action, India Office, Innov8 Old Fort Saket District Mall, Saket District Centre, Sector 6, Pushp Vihar, 110017 New Delhi, India; Department of International Health, Johns Hopkins Bloomberg School of Public Health, 615 N. Wolfe Street, Baltimore, MD 21205, USA; Division of Public Health Medicine, Public Health and Family Medicine, University of Cape Town, Observatory 7925, Cape Town, South Africa

**Keywords:** Cognitive interviewing, survey research, validity, methodological innovation, qualitative research

## Abstract

Cognitive interviewing is a qualitative research method for improving the validity of quantitative surveys, which has been underused by academic researchers and monitoring and evaluation teams in global health. Draft survey questions are administered to participants drawn from the same population as the respondent group for the survey itself. The interviewer facilitates a detailed discussion with the participant to assess how the participant interpreted each question and how they formulated their response. Draft survey questions are revised and undergo additional rounds of cognitive interviewing until they achieve high comprehension and cognitive match between the research team’s intent and the target population’s interpretation. This methodology is particularly important in global health when surveys involve translation or are developed by researchers who differ from the population being surveyed in terms of socio-demographic characteristics, worldview, or other aspects of identity. Without cognitive interviewing, surveys risk measurement error by including questions that respondents find incomprehensible, that respondents are unable to accurately answer, or that respondents interpret in unintended ways. This methodological musing seeks to encourage a wider uptake of cognitive interviewing in global public health research, provide practical guidance on its application, and prompt discussion on its value and practice. To this end, we define cognitive interviewing, discuss how cognitive interviewing compares to other forms of survey tool development and validation, and present practical steps for its application. These steps cover defining the scope of cognitive interviews, selecting and training researchers to conduct cognitive interviews, sampling participants, collecting data, debriefing, analysing the emerging findings, and ultimately generating revised, validated survey questions. We close by presenting recommendations to ensure quality in cognitive interviewing.

**Key messages**

- Cognitive interviewing seeks to bridge linguistic, social, and cultural gaps between the researchers who develop surveys and the populations who complete them to improve the match between research intent and respondent interpretation.
- This methodology is gaining prominence in global public health but could benefit from wider discussion and application in our field; to this end, we present a description of cognitive interviewing, its value and distinguishing features, and practical guidance for its application.
- The methodological musing calls attention to the practical details of its application, including researcher training, allocating adequate time for cognitive interviews, structured debriefs, and systematically revising and testing multiple iterations of the survey tool.

## Introduction

This methodological musing calls attention to cognitive interviewing, a qualitative research methodology for improving the validity of quantitative surveys that has often been overlooked in global public health. Cognitive interviewing is ‘the administration of draft survey questions while collecting additional verbal information about the survey responses, which is used to evaluate the quality of the response or to help determine whether the question is generating the information that its author intends’ (Beatty and Willis, 2007). This methodology helps researchers see survey questions from the participants’ perspectives rather than their own by exploring how people process information, interpret the words used and access the memories or knowledge required to formulate responses (Drennan, 2003).

Cognitive interviewing methodology emerged in the 1980s out of cognitive psychology and survey research design, gaining prominence in the early 2000s (Beatty and Willis, 2007). Cognitive interviewing is widely employed by government agencies in the preparation of public health surveys in many high-income countries [e.g. the Collaborating Center for Questionnaire Design and Evaluation Research in the Center for Disease Control and Prevention (CDC)/National Center for Health Statistics (2014) and Agency for Healthcare Research and Quality in the Department of Health and Human Services (2019) in the USA and the Quality Care Commission (2019) for the National Health Service Patient Surveys in the UK]. Applications in the global public health space are emerging, including to validate measurement tools undergoing primary development in English and for use in English [e.g. to measure family response to childhood chronic illness (Knafl *et al.*, 2007)]; to support translation of scales between languages [e.g. to validate the London Measure of Unplanned Pregnancy for use in the Chichewa language in Malawi (Hall *et al.*, 2013)] and to assess consumers’ understanding and interpretation of and preferences for displaying information [e.g. for healthcare report cards in rural Tajikistan (Bauhoff *et al.*, 2017)]. However, this methodology remains on the periphery of survey tool development by university-based academic researchers and monitoring and evaluation teams working in global health; most surveys are developed, translated and adapted without cognitive interviews, and publications of survey findings rarely stipulate that cognitive interviews took place as part of tool development processes.

Box 1. **The need for cognitive interviewing: examples from developing a tool to measure respectful maternity care among rural women in central India**

**Context: respectful maternity care in rural central Indi** **a**

We used cognitive interviewing to examine survey questions for rural central India, adapted from validated instruments to measure respectful maternity care used in Ethiopia, Kenya and elsewhere in India. This process illuminated extensive cognitive mismatch between the intent of the original questions and how women interpreted them, which would have compromised the validity of the survey’s findings (Scott *et al.*, 2019). Two examples are provided here.

**Cognitive interviews revealed that hypothetical questions were interpreted in unexpected way** **s**

A question asked women whether they would return to the same facility for a hypothetical future delivery. The researchers intended the question to assess satisfaction with services. Some women replied no, and, upon probing, explained that their treatment at the facility was fine but that they had no intention of having another child. Other women said yes, despite experiencing some problematic treatment, and probing revealed that they said this because they were too poor to afford to go anywhere else.

**Cognitive interviews revealed that Likert scales were inappropriat** **e**

The concept of graduated agreement or disagreement with a statement was unfamiliar and illogical to respondents. Women did not understand how to engage with the Likert scales we tested (5-, 6- and 10-point scales, using numbers, words, colours, stars, and smiley faces). Most respondents avoided engaging with the Likert scales, instead responding in terms of a dichotomous yes/no, agree/disagree, happened/did not happen, etc., despite interviewer’s attempts to invite respondents to convert their reply to a Likert response. For example, when asked to respond on a 6-point Likert scale to the statement ‘medical procedures were explained to me before they were conducted’, a respondent only repeated ‘they didn’t explain’. Other respondents, when shown a smiley face Likert scale, focused on identifying a face that matched how they felt rather than that depicted their response to the statement in question. For example, when asked to respond to the statement ‘the doctors and nurses did everything they could to help me manage my pain’, a respondent pointed to a sad face, explaining that although the doctors and nurses helped her, since she was in pain her face was ‘like this’ (i.e. sad). Without cognitive interviews, survey enumerators would unknowingly record responses unrelated to the question at hand or would attempt to fit respondent dichotomous answers into Likert scales using whatever interpretation the enumerator saw fit.

Cognitive interviewing recognizes that problems with even one detail of a survey question can compromise the validity of the data gathered, whether it is an improper word, confusing phrasing, unfamiliar concept, inappropriate response option, or other issue. Without cognitive interviews, gaps between question intent and respondent interpretation can persist, severely compromising the quality of data generated from surveys (Box 1). Furthermore, cognitive mismatch is often impossible to detect after data collection. Instead, responses recorded in the survey are taken as ‘true’, regardless of whether the respondents understood and answered the question in the intended manner and regardless of the assistance, adjustment, or interpretation provided by enumerators.

In this article, we argue that cognitive interviewing should be an essential step in the development of quantitative survey tools used in global public health and call attention to the detailed steps of applying this method in the field. We start by reviewing what cognitive interviewing is and consider the varied definitions and use cases in survey tool development. We next outline the recommended steps in survey tool development and then provide an overview of how to go about cognitive interviewing. We close by reflecting on the broader implications of cognitive interviewing.

## Defining cognitive interviewing

Cognitive interviewing enables researchers to assess how participants process information to comprehend and respond to survey questions. Cognitive interviews reveal and help correct issues including word choice, syntax, sequencing, sensitivity, response options, and resonance with local world views and realities (Table 1). These factors individually and collectively play a vital role in determining what cognitive domains a respondent accesses and whether these domains align with the construct that researchers are seeking measure. Examples for this paper have been drawn from cognitive interview data collected in rural India for purposes of developing survey tools to assess women’s experiences during pregnancy and childbirth (Scott *et al.*, 2019), as well as measure a variety of reproductive, maternal and child health outcomes including infant and young child feeding (IYCF) and family planning (FP) (Lefevre *et al.*, 2019).

**Table 1.** Components of survey tools assessed by cognitive interviewing

Survey tool component assessed	Explanation	Example
Word choice	Words used in the survey questions may not be understood by respondents, may have unintended alternative meanings, may be overly vague or specific or may be less natural than alternative words	When translating surveys from English to Hindi, we found that professional translators and Hindi-speaking researchers with experience in rural areas often selected formal Hindi words that were unfamiliar to rural women
Syntax	Sentences in survey questions may be too complex or too long, reducing respondent capacity to retain key features of the question	The question ‘During your time in the health facility did the doctors, nurses, or other health care providers introduce themselves to you when they first came to see you?’ contained too many words and clauses. By the time the researcher finished reading it, the respondent lost track of the core question
Sequencing	The order of questions may be inappropriate. Placing sensitive or emotionally charged questions too early in the survey can be uncomfortable for respondents and damage respondent–enumerator rapport, reducing the likelihood of a respondent providing a truthful and complete response	A survey on respectful maternity care initially asked post-partum women if they were verbally or physically abused during childbirth within the first few survey questions, to ensure that this crucial question was answered before any respondent fatigue set in. However, cognitive interviews revealed that women were uncomfortable with the question and unlikely to disclose abuse without first establishing rapport through a range of less emotionally intense questions
Sensitivity	Questions or response options may be too direct or include topics that are insufficiently contextualized, leading to respondent and enumerator discomfort and eroding rapport	When asking women about their birth companions, they found it strange and uncomfortable to be probed about whether male family members were with them
Response options	Response options may be insufficient to capture the actual range of responses or may be incomprehensible or uncomfortable for respondents	Likert scales with more than three response options were incomprehensible to most rural Indian women we interviewed. Asking women to estimate the amount of food they gave their child in the 24-hour dietary recall in terms of cups or bowls was considered illogical since roti (flatbread), a common food, does not fit into cups
Resonance with local worldviews and realities	Questions may ask about domains of importance to the research team but that do not resonate with respondent views or realities	‘Being involved in decisions about your health care’ is a domain of global importance in respectful maternity care. However, in rural India, the concept of healthcare workers involving the patient in healthcare decisions was unfamiliar and, when explained, considered undesirable
Cognitive mismatch	Questions may access respondent cognitive domains that do not map on to the domains intended by the researchers	Women were asked whether they would recommend the place where they gave birth to a friend, as a proxy for quality of care. However, women frequently responded ‘no’ because they did not have friends, did not want to tell other women what to do or did not think they should make recommendations for other people—which was unrelated to their maternity care experiences
Memory	Questions or response options may seek to access respondent memories in ways that are too cognitively demanding	Recalling specific post-partum practices from many months ago may not be possible for some respondents

While it is usually possible to identify and remedy linguistic and syntax issues in survey questions, cognitive interviewing cannot always solve deeper problems with survey research. Cognitive interviews may illuminate question failures arising from a mismatch between the underlying concepts that the survey attempts to measure and the concepts that resonate with the respondent’s worldview and reality (Schuler *et al.*, 2011; Scott *et al.*, 2019). In these cases, question revision will not achieve cognitive alignment between researcher and participant. Instead, researchers must drop questions from the survey and potentially generate new items.

There are many terms and approaches used for strengthening surveys, some of which may encompass cognitive interviewing or include components of it without applying the label (Table 2). We argue however, that cognitive interviewing should be a standalone approach integrated into a larger process of survey tool development.

**Table 2.** Approaches to strengthening surveys

Approach	Description	Comparison to cognitive interviewing	Issue
Expert review	Subject area experts review the survey tool and judge how well each questionnaire item truly reflects the construct it is intended to measure	An important form of validation but provides no insight into respondent understanding and interpretation of the survey questions	Experts are unable to predict how the survey respondents will interpret the questions
Respondent-driven pretesting	A small group of participants with the same characteristics as the target survey population complete the survey. Researchers elicit feedback during the survey or at the end through debriefings. Feedback elicitation can include targeted probes about questions that appeared problematic, in-depth exploration of each question, probing on a random sub-set of questions, or asking participants to rate how clear the question was	Respondent-driven pretesting may overlap with cognitive interviewing (e.g. eliciting in-depth reflection on how the participants interpret questions and formulate answers as they proceed through the survey) However, it may also differ from cognitive interviewing by focusing instead on post-survey reflections through ratings or group debriefs (Ruel, Wagner and Gillespie, 2016)	Low methodological clarity: can be the same as cognitive interviewing or quite different
Translation and back translation	After translating a survey from the origin to the target language, a different translator ‘blindly’ translates the survey back. Differences are then compared and resolved (Weeks, Swerissen and Belfrage, 2007)	Back translation includes the same close attention to language and meaning as cognitive interviewing However, it does not examine cultural appropriateness or the extent to which questions achieve cognitive match between researchers and respondents	Involves bilingual translators whose world view and experience do not match the target population’s, making them unable to comment on the tool’s appropriateness
Pilot testing	Enumerators administer the survey to a small group of participants with the same characteristics as the target survey in as close to real world conditions as possible	Pilot testing explores survey length, modality (e.g. is the computer assisted personal interviewing (CAPI) programming and tablet hardware functioning properly?), and skip patterns, and catches obvious problems with content and translation. Pilot testing is undertaken by members of the quantitative enumeration team who will conduct the survey at scale and focuses on the practical application of the survey questions. One pilot test goes through the whole survey tool with a sample participant Cognitive testing is undertaken by specially trained qualitative researchers with a focus on extensive probing to understand the cognitive process underlying each response provided. One cognitive interview goes through a curated sub-set of questions from the survey tool with a sample participant Cognitive interviewing is not optimal for exploring survey length, modality and skip patterns, but involves in-depth exploration of the resonance of content with local worldviews, and close attention to vocabulary, syntax, response options, question style, and conceptual nuance	Focuses on the mechanics of implementation while cognitive testing focuses on the survey questions achieving shared understanding between researcher intent and respondent interpretation

## Fitting cognitive interviewing into larger survey tool development

**Figure 1.**
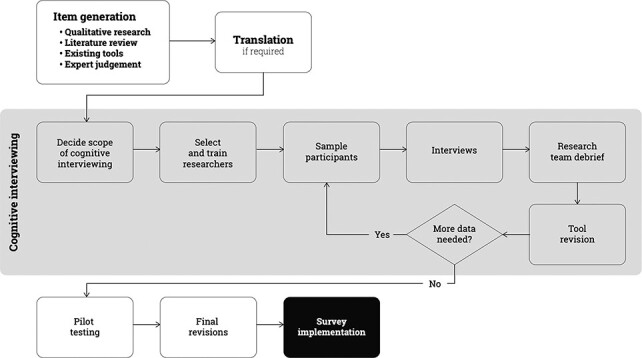
Situating cognitive interviewing within the larger process of tool development

Survey tool development starts with item generation, which may include a variety of approaches, including in-depth interviews with respondents, review of literature and existing survey tools, and expert review. This is followed by translation, cognitive interviewing, content modification, and then pilot testing (Figure 1).

Box 2. **Overview of the Kilkari evaluation**

**What is Kilkari?** Kilkari is India’s flagship direct-to-beneficiary messaging programme. Pregnant and post-partum women receive one weekly phone call containing a short (1.5 minute) pre-recorded health message on topics including preparing for childbirth, caring for newborns, IYCF, and FP.

**Kilkari evaluation:** The Kilkari evaluation (Lefevre *et al.*, 2019) was a randomized controlled trial in rural Madhya Pradesh, India. In 2018, 5095 pregnant women were enrolled and randomized to receive Kilkari or not. An endline survey in 2020, when the study participants were 12–17 months post-partum, assessed whether receiving Kilkari changed women’s knowledge or practice.

**Endline Kilkari evaluation survey tool:** The draft endline survey included 12 modules to assess study participants’ knowledge and self-reported practice on topics covered by Kilkari, as well as information on socio-economics, decision-making power in the household, interaction with community health workers, exposure to Kilkari, and media consumption patterns. Draft questions were drawn from a mix of tools identified in the literature, including the Demographic and Health Survey and Multiple Indicator Cluster Surveys, and developed by other academic teams.

## Steps in undertaking cognitive interviewing

Greater uptake of cognitive interviewing and explicit description of the process would be a strong contribution to improving the validity of survey research in this field. In this section, we discuss the steps in conducting rigorous cognitive interviews: defining scope, selecting researchers, training, sampling, data collection, and analysis. We draw illustrative examples from our experience with cognitive interviews to refine survey content for the Kilkari evaluation (Box 2).

### Defining the scope of cognitive interviews

In an ideal scenario, almost all questions in a data collection tool would be tested. However, time and available resources often limit how much of the survey can be tested. Examining a survey question during a cognitive interview takes far longer than asking the same question during the field survey itself. In a cognitive interview, each survey question must first be asked and answered in a quantitative manner and then discussed in an in-depth qualitative manner through a series of probes to determine how the respondent interpreted the question.

Multiple cognitive interview guides can be developed to examine sub-components of the survey questions. Thus, a cognitive interview guide can be developed to assess one portion of the survey’s questions with one set of participants, while a second interview guide can be developed to assess a different set of questions from the survey with a different set of participants, and so on. But even with multiple cognitive interview guides, researchers will likely still have to prioritize a sub-sample of questions. Selecting which questions to test is a judgement decision that can be guided by focusing on the questions most central to measuring the key outcomes of interest and the questions that are new, conceptually complex, or have never been applied to this respondent population. It is also important to keep blocks of questions (e.g. subject modules) together since they build on and relate to one another. Box 3 presents an illustrative example drawn from our team’s process of defining the scope of cognitive interviews in the Kilkari evaluation.

Box 3. **Defining the scope of cognitive interviewing for Kilkari endline survey**

**Priority areas of the tool selected for CI:** The draft endline survey tool contained 180 questions and was to take 90 minutes to administer in the field, at about 30 seconds per question. Although we wanted to test each question, it was not feasible to do so. While the time required to test each question in the cognitive interview varies widely, we found it appropriate to allocate each draft survey question at least 3 minutes in the cognitive interview: about 30 seconds to simply ask the question and attempt to record an answer mimicking the survey data collection, and then an additional 2.5 minutes for cognitive probing. Attempting to test each question of a 180-question long survey would require an (impossibly long) nine-hour cognitive interview.

**Figure 2.**
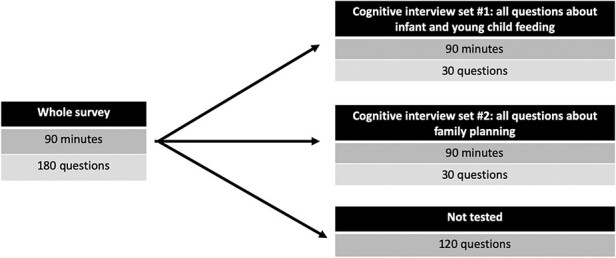
How much of the survey can you test through cognitive interviews?

Since the Kilkari evaluation’s priority outcomes were infant and young child feeding and the use of modern contraception, we focused on cognitive interviews for the questions on these topics. We went through the draft survey tool and identified all the questions on these two topics, which were spread across modules on knowledge, practice, decision-making power and discussion. There were approximately 60 questions, which would still take 3 hours to cover in one cognitive interview. We thus decided to split them into two separate sets (Figure 2).

Even with limiting the number of survey questions in our cognitive testing guide to just 30, many interviews still had to wrap up before completing all 30 questions. Sometimes respondents had to leave early or were distracted. Many times the researchers found that initial questions took longer than anticipated and thus had to end the interview before completing the guide. This was particularly the case with the least educated respondents and the earlier draft version of questions. In these cases, participant comprehension was very low and thus the researchers spent a long time explaining questions to participants and seeking to understand the various ways in which questions failed.

**Key learning:** You can test far fewer questions in a cognitive interview than you can cover in a survey of comparable duration (usually 1.5 hours). Multiple cognitive interview sets may be required to test all priority survey questions.

### Selecting and training researchers to conduct cognitive interviews

Cognitive interviewing requires high-level analytical understanding, linguistic insight, and collaboration among researchers. Researchers must be fully proficient in the language spoken by the target population, as well as the original language of the draft questions, in cases where translation is involved. This proficiency is vital so that each researcher understands the nuances of the original questions and can carefully adapt the phrasing of the questions to ensure local understanding. The effects of similarities and differences in interviewer/participant’s gender, age, sexuality, class, and ethnicity have been considered in the qualitative research methodological literature (Fontana and Frey, 1994; Hsiung, 2010). For cognitive interviewing, the same considerations apply, wherein interviewer identity must be considered in light of the topic being studied and the research context (Hsiung, 2010). Ideal researchers are strong qualitative interviewers, so that they can undertake appropriate probing and use verbal and nonverbal approaches to setting respondents at ease and handling respondent discomfort with the potentially unfamiliar process of the cognitive interview. They should also be familiar with quantitative survey research, so that they can understand how quantitative enumerators will administer questions and seek to determine appropriate answers. Box 4 discusses team composition for the Kilkari endline survey cognitive interviews.

Box 4. **Selecting researchers for the Kilkari endline survey cognitive interviews**

**Team structure:** Our eight-person research team consisted of five female researchers, a male research team manager, a male logistics coordinator and a female team lead. The five researchers were all master’s level social scientists with prior qualitative research experience and training. The researchers worked in pairs, with the fifth and most junior researcher providing backup support. The research manager worked with the logistics coordinator to handle day-to-day logistics, sampling and data management. They also handled sensitive community relationship issues, such as taking curious onlookers (particularly husbands, who were sometimes keen to jump in and answer questions for their wives) away from the cognitive interview. The research lead and the research manager conducted training, developed the research protocol, and ran debriefs.

**Profile and selection of researchers:** Researchers were fluent in Hindi and English, had prior qualitative experience and where possible, and had worked previously in Madhya Pradesh. Additional experience with and sensitivity to key issues relevant to successful qualitative interviewing in rural India included: awareness of gender and caste power dynamics; knowledge of rural Indian community dynamics including health system dynamics; capacity to effectively probe; and understanding of rapport-building.

**Key learnings:** Experienced researchers, ideally with an understanding of both quantitative and qualitative data collection, are required and should work in pairs (one to lead the interview and one to take detailed notes). Fluency in the survey’s starting language and target language are vital in cases where the survey is translated.

Box 5. **Training researchers for the Kilkari endline survey cognitive interviews**

**Training:** Training was composed of the following modules:

1. Overview of the entire Kilkari evaluation;
2. Overview, objectives and sampling for cognitive testing within the Kilkari evaluation;
3. Principles of cognitive interviewing;
4. Findings from earlier cognitive interviewing on other topics to showcase the types of issues identified through this research process and how these cognitive failures were resolved to strengthen another survey tool;
5. Principles of qualitative interviewing;
6. Research ethics and consent processes;
7. Data management, cover sheets, field logistics, and safety;
8. In-depth lecture and discussion on research topic 1: infant and young child feeding including recommended practices on exclusive breastfeeding, what exclusive breastfeeding means, and recommended practices on complementary feeding;
9. Question-by-question examination of the cognitive interview guide on infant and young child feeding (IYCF) to ensure that each researcher understood the underlying intent of each question;
10. Role play to practice cognitive interviewing;
11. In-depth lecture and discussion on research topic 2: family planning;
12. Question-by-question examination of the cognitive interview guide on family planning to ensure that each researcher understood the underlying intent of each question.

Modules 9 and 12 required two days each. We examined each survey question to be tested in the field—including the answer options—to thoroughly understand the question’s intent, assess the Hindi translation, and hypothesize potential areas of confusion that could arise. We also examined the pre-developed cognitive probes for each survey question and edited them where necessary (more on this in Box 7). We furthermore discussed how we could handle potential participant reactions to the questions.

We covered Modules 1–10 in the first week-long training. We then conducted our data collection on IYCF. Only once that data collection process was complete did we proceed to Modules 11 and 12. In separating exposure to the two different cognitive interview topics and guides, we ensured that the team was immersed in and focused on only one area at a time, and did not forget the family planning content while working on IYCF. While in many cases piloting is indicated, the richness of initial data exceeded expectations and the resulting interviews were included in final analyses.

**Key learnings:** Some researchers initially struggle to understand the intent of cognitive interviewing and struggle to wear two hats; they must toggle between ‘quantitative enumerator mode’ wherein they read the question as it is written and attempt to elicit a response as if conducting a quantitative survey, and ‘qualitative interviewer mode’ wherein they explore what the respondent was thinking about and draw from narrative explanations to access memories or opinions. Role play during training and close observation of the interviews is necessary to ensure the research team truly understands the intent of cognitive interviews and are capable of implementing this data collection methodology.

Given the complexity of conducting cognitive interviews and the likelihood that the method will be new to members of your research team, close attention to training the cognitive interview research team is vital (Box 5). Even experienced researchers are likely to be unfamiliar with this research methodology and require time to fully understand the macro-level intent of the data collection and the micro-level data collections strategies required. The interviewers must also be well versed in the topic(s) being studied as well as the cognitive intent of each to formulate appropriate emergent probes (Willis, 2005). For instance, if a survey is studying family planning and includes a section to determine which methods of contraception a participant has heard about, the interviewer herself must know the difference between injectable contraceptives and implanted contraceptives. Only then can the interviewer to appropriately probe to find out what a participant was referring to by ‘that one in the skin’. In-depth training and strong oversight for researchers new to cognitive interviewing can ensure that they do not become sidetracked by focusing on recording the participant’s answer to the survey question rather than exploring the participant’s interpretation.

### Participant sampling

Participants in cognitive interviews must be drawn from the same profile as the intended survey respondents. It is difficult to predict how many participants will need to be sampled in order to capture all the cognitive failures with the survey questions. A relatively small number of well-conducted cognitive interviews can yield an enormous amount of rich information, particularly when there is a large cultural and linguistic gap between the researchers and respondent population. Our research has found reasonable evidence of saturation with a total of 20–25 participants over three rounds, which broadly aligns with recommendations from high-income countries, such as the US Center for Disease Control and Prevention’s guidance of 20–50 respondents (CDC/National Center for Health Statistics, 2014) and lead American cognitive interviewing methodologist Gordon Willis’s 8–12 subjects per round, multiplied by 1–3 rounds (Willis, 2014). Direct comparison of sample sizes across studies is often inappropriate because they involve testing different numbers of questions over different numbers of rounds, with varying types of respondent groups, and some involve translation while others do not. Published literature highlights a range of sample sizes, including 10 urban Greenlandic residents for a sexual health survey available in three languages (Gesink *et al.*, 2010); 15 people per language group per round (four languages, two rounds and total of 120 interviews) for a women’s empowerment in agriculture survey in Uganda (Malapit, Sproule and Kovarik, 2016); 24 people across seven ethnic groups for a mental health survey in the UK without translation (Care Quality Commission, 2019); 34 people stratified for age, gender, education level and location in rural Bangladesh for assessing the cultural suitability of a World Health Organization (WHO) quality of life assessment (Zeldenryk *et al.*, 2013); 20 women and 20 men to improve a healthcare report card in Tajikistan (Bauhoff *et al.*, 2017); and 49 women in rural Ethiopia to assess the resonance of a WHO question on early initiation of breastfeeding (Salasibew *et al.*, 2014). The following three suggestions can help guide sampling in an efficient and rigorous manner.

First, focus on sampling participants from within the survey’s target population who are most likely to struggle with the survey; these are usually the least educated and most marginalized people (Box 6). Interviewing these participants will reveal the weaknesses of the survey most rapidly and create the opportunity to adapt the survey to be comprehensible to the entire target population. While some researchers recommend engaging a range of respondents (Willis, 2005), our experience in rural India found that participants with higher than average education and exposure were far less useful in identifying issues than participants with the lowest education and exposure.

Box 6. **Sampling for the Kilkari endline survey cognitive interviews**

**Cognitive interview population:** Since the endline survey would be conducted among the 5095 women enrolled in our evaluation 1–1.5 years after they gave birth, our cognitive interviews were conducted among participants with a similar profile: rural women in Madhya Pradesh who had access to mobile phone and who are mothers to a child between 12 and 17 months in age. Within this profile, we skewed our sample towards women with low levels of education, from marginalized castes, and in lower socio-economic strata.

**Sample:** We conducted two sets of cognitive interviews: one on IYCF (*n* = 21) and one on family planning (FP) (*n* = 24) (Table 3). Each set required three rounds of interviews, with the questions revised twice: after Round 1 and after Round 2. Round 1 and Round 2 involved a higher number of respondents because we sought sufficient data to understand and document the range of cognitive failures associated with the draft survey questions. Round 3 required fewer respondents because by that stage we were generally confirming that the questions were working as intended. Concentrating on lower literacy and marginalized women was highly efficient at exposing problems with the draft questions and enabling us to reduce the number of interviews necessary. With family planning, we set out to test a portion of questions that were to be asked only of women who had become pregnant in the year since the birth of their previous child. However, this event was relatively rare and we considered it inappropriate to screen women at enrolment for this event, so we oversampled in hopes of including at least a few women who would not skip out of this portion of questions.

**Table 3.** Sample of participants for cognitive interviewing in Kilkari

Topic of survey questions	Round 1	Round 2	Round 3	Total
Set 1: IYCF	7	8	6	21
Set 2: FP	13	6	5	24


**Key learnings:** We found that as few as six respondents per round was sufficient to expose cognitive failures and enable revision for the next iteration. Focus on sampling the respondents who are most likely to struggle to comprehend the survey questions—generally those within your target population who have the lowest exposure or education. You can probably conduct an unlimited number of cognitive interviews and continue identifying small potential improvements. However, returns diminish—we found three rounds to be sufficient.

Second, think about sampling in terms of iterations. Cognitive interviewing requires an initial round of interviews using the first version of the survey. Then, the team will revise the survey based on detailed debriefing and take version two to the field for another round of cognitive interviews. Additional rounds of revision are required until the survey achieves cognitive match between researcher intent and participant comprehension (Figure 1). The number of respondents required may reduce from iteration to iteration as the survey questions become increasingly more appropriate to the local context.

Third, aim to include participants whose experiences exhaust the domains covered by the survey (Beatty and Willis, 2007). If all of your participants skip out of a certain section of the survey, you will not be able to test the questions in this section. Ideally, your recruitment strategy can pre-identify people who will complete specific sections of the survey. However, if it is difficult to pre-identify respondents who have experienced specific domain of interest that have low prevalence, you will have to increase your sample size.

### Data collection

Cognitive interviewing begins with first asking the original survey question exactly as it is written and recording the respondent’s answer using the original response options. The interview then proceeds by eliciting feedback from the respondent to understand how they interpreted the question and why they gave the response provided. Two main approaches have been used for eliciting this feedback: (1) probing and (2) ‘think aloud’ (Table 4).

**Table 4.** Approaches to eliciting feedback in cognitive interviews

Approach	Description	Benefits	Drawbacks
Think aloud	Participant talks through their mental processes and memory retrieval as they interpret questions and formulate answers (Knafl *et al.*, 2007)	Lower risk of interviewer biasing responses Interviewer does not need much training (Willis, 2005)	High cognitive burden on participant Confusing for many participants, particularly poorer and less educated participants, who are unfamiliar with idea of reflecting and articulating thoughts Can be embarrassing for participants who do not understand the request Does not allow for targeted exploration of areas of researcher’s interest (Miller, 2003; Zeldenryk *et al.*, 2013)
Probing	***Scripted*:** Researcher uses pre-developed questions to interview participant about their interpretation of the question, such as ‘what does [word] mean to you?’ or ‘why did you say [answer]?’ (Weeks, Swerissen and Belfrage, 2007) ***Emergent*:** Research formulates probes during the cognitive interview to explore emergent issues, such as ‘Earlier you said you had never done [activity] but now you said you had completed [sub-activity]. Why did you say that?’ (Beatty and Willis, 2007; Zeldenryk *et al.*, 2013)	Lower burden on respondent More comfortable and natural for respondents to answer interview questions than articulate their thought processes Enables researcher to explore inconsistencies and seemingly illogical responses (Miller, 2003; Zeldenryk *et al.*, 2013)	May be misinterpreted by respondents as an examination or a test of their knowledge, vocabulary skills, or cognitive abilities, thus leading to respondent withdrawal or nervousness Emergent probing in particular requires that the researcher has complete familiarity with the topic being studied as well as the intent of each survey question and overall flow and content of the entire survey so that they can track meaning and make connections across the survey questions

Probing requires that enumerators use a combination of scripted and unscripted prompts to guide the directionality of the interview, while ‘think aloud’ asks the respondent to verbalize their thoughts while interpreting the question and formulating their response (Willis, 2005). While some researchers find success incorporating the think aloud approach (Ogaji *et al.*, 2017), probing is gaining consensus as the ideal method (Willis, 2014) and is particularly appropriate in global public health when working with respondents who find it easier to answer questions than verbalize their thought process (Zeldenryk *et al.*, 2013). Even with probing, careful effort must be made to manage the shortcomings of this method. Researchers must provide clear explanation that the exercise seeks participant feedback on the questions and that any confusion or incomprehension is entirely the research team’s fault, not the participant’s. Box 7 provides an example cognitive interview question and reflections on the data collection process.

Box 7. **Data collection: cognitive interviews for Kilkari endline survey**

**Figure 3.**
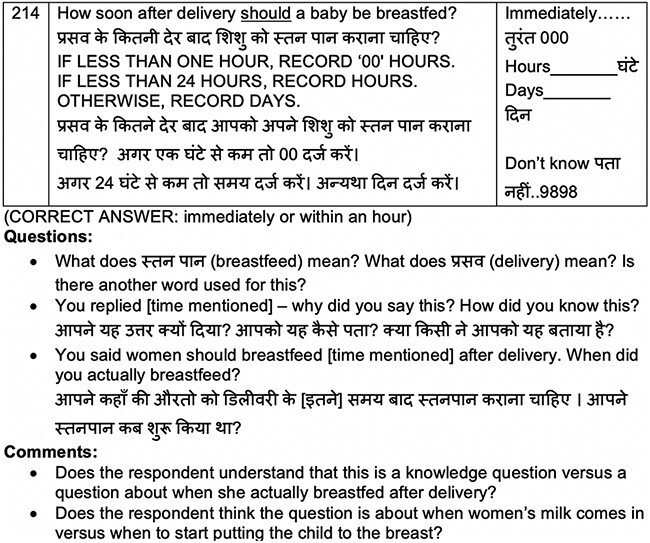
Example question from the IYCF cognitive interview guide


**Data collection tool:** The data collection tool consisted of each draft survey question (and its answer options) followed by suggested scripted probes and comments about areas to explore. Researchers were also strongly encouraged to use emergent probes based on specific information that arose during their interview. Figure 3 provides an example question from the IYCF cognitive interview guide.

**Data collection experience:** We found that some participants struggled to understand the purpose of the interview and felt embarrassed or annoyed by the probes. Some researchers initially struggled to move beyond the scripted probes, at times failing to probe on issues that arose and demanded attention. During debriefs, we dissected each question and subsequent probing and emphasized the importance of unscripted probing to better understand respondent’s cognitive processes. For instance, if a respondent said that a baby should be breastfed 3 hours after birth, and we knew from other questions in the survey that the respondent had a caesarean delivery, the interviewer had to probe to determine whether the participant experienced delayed breastfeeding due to her post-operation recovery and whether her reply on when babies should breastfeed was actually her description of when her baby was breastfed. For another example, if a participant said she had not heard of condoms when directly asked in the knowledge section and then later said that she had used condoms when asked about her use of birth control, the researcher had to have the insight to circle back to the initial knowledge question about condoms to determine if the initial response was driven by low comprehension (maybe we used an unfamiliar word for condom) or discomfort (shyness) or another factor.

**Key learnings:** Researchers need deep familiarity with the tool and subject matter to successfully combine scripted and emergent probing. Rapport must be developed with the research participants and they must be regularly reassured and reminded that any confusion caused by the survey questions is the research team’s ‘fault’ and not theirs.

Data collection ideally requires two researchers to conduct every interview: one to lead the questioning and one to take responsibility for notetaking and to support the questioning. While in some qualitative interviews junior researchers can serve as appropriate notetakers, for cognitive interviewing experienced, trained researchers should perform this role. Notetaking is essential in cognitive interviewing because debriefs and rapid revision of survey questions depend on detailed notes being available for analysis after the interview. Notetakers must be trained to record the various forms of cognitive failure that occur during the interview as well as non-verbal feedback from the respondent. While recordings of the interviews can also be reviewed during the debriefs, reviewing the whole audio file or generating and reading a complete transcript requires far more time than is available in the field. High-quality notes are the backbone of the rapid field-based analysis, discussed next, that enables production of revised survey questions.

### Debriefs and analysis

To ensure the robustness of findings, cognitive interviewing requires alternating data collection with team debriefing sessions from which revisions to the tool may emerge. This process is iterative with resulting changes to language and tool content requiring additional testing until the final round of cognitive interviews showcase high comprehension amongst respondents.

Box 8 showcases the field work approach from the Kilkari endline survey cognitive interviewing, which found that 1.5 days of debriefing time was required for every 7–8 interviews conducted. For every survey question being tested, researchers will need to budget at least 3 minutes during the interview and an hour during the debrief to discuss what each respondent said for that question and consider how to improve the question. Simple questions that work well take the least amount of time—perhaps less than 3 minutes during the interview to establish that the respondent interpreted the question as expected and another 10 minutes during the debrief to clarify that all interviews had similar success. Questions with translation problems demand more time. Questions with deeper conceptual issues, such as entire concepts failing to resonate with respondent worldview, take the most time. The schedule for data collection and the steps used in debriefing are discussed in Box 8.

Box 8. **Debriefing and analysis for the Kilkari endline survey cognitive interviewing process**

**Phase 1. Revising the survey tool:** We aimed to conduct approximately six cognitive interviews in one day of fieldwork. After collecting data through about eight interviews on the first version of the instrument, we required 1.5 days for debrief and revision before returning to the field to test the second iteration of the tool (Table 5).

**Table 5.** Illustrative schedule of 1 month of cognitive interview field work

Day 1	Day 2	Day 3	Day 4	Day 5	Day 6	Day 7
Training, including topic lecture and discussion on IYCF and detailed review of the IYCF cognitive interview guide						Break
**Day 8**	**Day 9**	**Day 10**	**Day 11**	**Day 12**	**Day 13**	**Day 14**
5 cognitive interviews (CIs) on IYCF version 1	Morning: 3 CIs on IYCF version 1 Afternoon: Debrief	Continue debrief and revise survey questions, create IYCF version 2	5 CIs on IYCF version 2	Morning: 2 CIs on IYCF version 2 Afternoon: Debrief	Continue debrief and revise survey questions, create IYCF version 3	Break
**Day 15**	**Day 16**	**Day 17**	**Day 18**	**Day 19**	**Day 20**	**Day 21**
6 CIs on IYCF version 3	Last debrief and revisions to create final version of IYCF questions	Topic lecture and discussion on FP and detailed review of the FP cognitive interview guide		6 CIs on FP version 1	7 CIs on FP version 1	Break
**Day 22**	**Day 23**	**Day 24**	**Day 25**	**Day 26**	**Day 27**	**Day 28**
Debrief and revise survey questions	Additional debrief and revision, create FP version 2	6 CIs on FP version 2	Debrief and revise survey questions, create FP version 3	5 CIs on FP version 3	Last debrief and revisions to create final version of FP questions	Break

Components of debriefs:

1. All researchers who conducted and took notes during the cognitive interviews attend, with the lead researcher serving as facilitator.
2. The debrief begins with collecting consent forms, uploading of audio recordings, and entering basic data information (assigning each interview a unique ID and documenting the duration, location, respondent age, etc.) into a data management system.
3. A spreadsheet is then used to document and systematize the debrief. Our team found success using rows to list the quantitative survey questions and response options and columns to list each interview by unique ID.
4. The team proceeds survey question by survey question. For each survey question, the researchers draw from the interview notes and occasionally from reviewing the audio recordings to document and discuss how the participant(s) that they interviewed responded to the question, what answer they provided (if any), and what additional information the cognitive probing elicited. The researchers and/or facilitator write extensive notes in the spreadsheet for each question to summarize what was said for each participant.
5. Revisions—rewording, rewriting, reordering, or removing questions—are then made by the entire team to attempt to resolve issues. These revisions are documented in the spreadsheet by adding a new column next to the original question.
6. After each question has been discussed, each interview participant’s responses have been shared, and each revision has been formulated in the spreadsheet, a revised cognitive interview guide is developed, with updated probes as needed.
7. The debrief closes with a discussion of research challenges, logistical considerations for the next day’s fieldwork, and participant sampling.

**Phase 2. Preparation of peer review manuscripts (optional)**

Often the end goal of cognitive interviewing is the production of a revised survey instrument that is valid and locally grounded. If this is the case, by the end of the final debrief, the team is finished. However, in cases where additional dissemination of the results of the cognitive interviewing is warranted, the cognitive interviews should be transcribed for analysis. When necessary, they also have to be translated, which demands extreme care. Significant portions of text in the original language must be retained to capture nuance in meaning around vocabulary words. The researchers should themselves carry out or at least check the translations. Thematic analysis can then be used to classify the text segments in the transcripts according to the cognitive failures exemplified.

**Key learnings:** Each cognitive interview generates an enormous amount of data that must be documented and used for subsequent revisions in the field. Cognitive interviewing data collection must be balanced with extensive time allocated for debriefing and revision.

### Support to quantitative survey training

Once the survey has achieved strong performance in the target population, cognitive interviewing is complete. The larger survey enumeration team will be formed and trained. It is useful to send some or all of the cognitive interview researchers to support the quantitative enumerator training and pilot testing. The cognitive interviewers can explain to the quantitative survey team why questions are worded and ordered the way they are and how to handle the types of responses that may arise in the population.

## Conclusion

The devil is in the details when it comes to cognitive interviewing—in terms of both the quantitative survey that this method hones and the cognitive interviewing method itself. Cognitive interviewing focuses on getting each detail of a survey question right to ensure valid data collection. Each word chosen, the exact syntax used, the response options provided (yes/no, Likert, etc.), the addition or removal of examples, the question styles selected (such as hypotheticals and true/false statements), and the resonance of the underlying constructs being assessed will all influence the alignment of researcher intent and respondent interpretation and response. The application of cognitive interviewing also demands careful attention to detail. Researchers must allocate adequate time and attention to each stage of the process and must ground difficult decisions in strong methodological logic. Cognitive interviewing demands tough decisions on which questions to test to ensure that the scope of the exercise is appropriate. The research team must be carefully selected and trained so that they can set respondents at ease and probe effectively to identify and document cognitive failures in real time. Research participants most likely to yield rich data must be sampled. The cycles of interviews, debriefing, analysis, and revision must be structured, meticulous and well documented.

As the methodology of cognitive interviewing continues to evolve in this field, the recommendations in Table 6 to ensure quality can help develop standards for research rigour.

**Table 6.** Recommendations to ensure the quality of cognitive interviews

Component	Recommendations	Rationale for recommendations
Scope of survey tool tested	The cognitive interview guide includes a reasonable number of survey questions to complete in 1.5 hours (likely around 30 questions) Additional guides should be developed if the total interview length exceeds 1.5 hours	The cognitive interview requires discussion time and probing about each survey question A 1- or 1.5-hour quantitative survey tool will require over 10 hours of cognitive interview time if each question is examined in the cognitive interview; therefore, a priority sub-set of draft survey questions must be selected or multiple guides developed and the sample expanded
Developing the cognitive interview guide	The cognitive interview guide includes survey questions, scripted probes and guidance on areas to explore through emergent probes	Researchers require guidance on which areas of the survey question to explore; they must also be encouraged to develop emergent probes throughout the course of the interview
Recruiting and training researchers	Researchers should be highly educated social scientists with prior qualitative research experience; quantitative survey research experience is desirable Researchers must be fluent in the local language, and, when relevant, the language of the broader team Ample time should be allocated for training, in order to cover: orientation to the purpose of the larger study, detailed instruction on cognitive interviewing, in-depth topic area teaching, question-by-question examination of the guides and role play	Cognitive interviewing is complex and quite different from mainstream qualitative research in terms of purpose, interview skills and debriefs. The field researchers drive the quality of the interviews and are fundamental in creating the final revised survey questions Linguistic issues are the most common problems with surveys that have been translated from English into regional languages. The researchers must be fluent in both languages in order to ensure nuance is captured across the translation, while adapting the language to local norms Extensive training is vital to orienting the researchers, including ensuring they understand the topics being assessed, the intent of each survey question, and how to carry out effective cognitive probing
Participant sample characteristics	Cognitive interview participants are from the same geographic area as the target respondent population Cognitive interview participants have similar socio-demographic characteristics to the survey target population Within the socio-demographic profile of the sample population, individuals with lowest levels of education, literacy and mobility and most marginalized should be prioritized	Cognitive interviewing enables local adaption and thus requires local participants who mimic the characteristics of the intended survey respondents Cognitive failures in the drafts survey questions are most efficiently and comprehensively identified by interviewing participants who are most likely to struggle with the material
Conducting interviews	Interviews should be carried out in pairs of trained qualitative researchers: one to conduct the interview and one to take notes throughout the interview	Notetaking is as important as leading the cognitive interview because debriefs and revisions of the survey questions depend on the notes taken during data collection. Notetakers must be as well trained and experienced as the interviewers
Debrief and analysis	Balance data collection with debriefing: Conduct a few (approximately 6 or 7) cognitive interviews and then allocate a day or more for debriefing and revision—do not gather a lot of data without time for reflection Multiple rounds of data collection should be conducted to test subsequent versions of the draft survey questions	Conducting a large number of cognitive interviews before pausing to debrief and revise the survey question is inefficient and impractical
Supporting quantitative survey enumerator training	Researchers who conducted the cognitive interviews should attend the survey enumerator training	Researchers who carried out the cognitive interviewing can explain the rationale for the final wording of the questions to the survey enumerations, provide locally grounded orientation to field realities (including local vocabulary) and help the enumerators anticipate the types of challenging responses they are likely to receive in the field

Survey research is fundamental to shaping our understanding of health systems. Surveys may aim to measure a range of outcomes, including population health, practices, care-seeking, attitudes towards services, and knowledge on health issues and topics. Researchers, practitioners, and policymakers rely on survey data to assess the scope of health or health system problems, prioritize the distribution of resources, and evaluate the effectiveness of programmes and interventions. It is thus crucial that researchers ensure that survey instruments are valid, i.e. that they truly measure what they intend to measure. Cognitive interviewing must be recognized as a fundamental validation step in survey development (Beatty and Willis, 2007; Willis and Artino, 2013), alongside literature review, expert consultation, and drawing from previously developed survey tools (Sullivan, 2011).

Ultimately, the need for cognitive interviewing in global public health arises from a gap, whether linguistic, cultural, or socioeconomic, between researchers and respondents. The greater this gap, the more space there is for cognitive mismatch to occur, leading to invalid research findings, and the more important cognitive interviewing will be in reducing this divergence. At the low-risk end of the spectrum are those with the smallest gap, such as surveys that are developed, administered, and analysed by the same population that completes them, as may occur during participatory action research. At the high-risk end of the spectrum with the greatest gap between researchers and respondents are surveys developed by researchers in one setting and adapted by another research group for use in a different setting, such as surveys developed in English in Western contexts and then translated to other languages and administered in non-Western settings. Cognitive interviewing may identify the most egregious survey question failures in the latter instance but can also identify surprising gaps between researcher intent and respondent interpretation even in the former.

## Data Availability

The data underlying this article will be shared on reasonable request to the corresponding author.
